# American Academy of Dental Science

**Published:** 1892-03

**Authors:** 


### AMERICAN ACADEMY OF DENTAL SCIENCE.

The regular monthly meeting of the American Academy of Dental Science was held at the Boston Medical Library Association rooms on Wednesday, December 2, 1891, President Brackett in the chair.

A paper by Forest G. Eddy, D.M.D., subject, "The Use of Gutta-Percha as a Root-Canal Filling," was read.

(For Dr. Eddy's paper, see p. 195.)

### DISCUSSION.

*Dr. Briggs.*—I am very glad to hear this encomium upon the use of gutta-percha as a root-filling. Ever since hearing a paper read some years ago by the late Dr. Whitten, on immediate root-filling, I have followed the plan which he then laid down. I believe he afterwards changed the filling material from gutta-percha to oxychloride of zinc, on account of the antiseptic properties of the latter substance. My experience, however, in treating severe cases with gutta-percha was so satisfactory that I thought it unnecessary to make a change.

I have nothing to add to the paper, and endorse it fully in all its details.

I want to speak more particularly of filling roots having fistulæ. In such cases it has been my custom to pump liquid gutta-percha through the root-canal, and follow it with solid gutta-percha until a line of liquid gutta-percha appeared at the fistulous opening. Where this plan has been followed cases have been particularly successful. I had a gentleman in my chair to-day for whom I

accomplished this result. About nine years ago he came to me for the treatment of the right inferior first and second molars, both teeth having fistulæ opposite their roots.

These fistulæ had been discharging for years. The roots had been filled according to another method by a very careful operator, but the teeth were loose as a result of their fistulæ. I drilled through the roots with a large drill, got a free opening, and filled them, so that the gutta-percha came out through the fistulæ as I have described. Of course the teeth were a little sore; but the gum healed and the teeth tightened up, and to-day the gum is perfectly normal, except that it is full of cicatrices.

That case was a type of a number of cases, which led me to feel that you must fill thoroughly,—not only the root itself, but often through the root,—with a substance compatible with the tissues, like gutta-percha. The tissues encyst it as they would a leaden bullet.

You may say that oxychloride of zinc would have the additional advantage of being an antiseptic; but the point is, to make the root aseptic before you begin to fill.

*Dr. Banfield.*—I would like to ask Dr. Eddy or Dr. Briggs whether, in an anæmic person, after gutta-percha is forced through the fistula, they would expect the tissues to heal as quickly as in a more healthy person?

*Dr. Eddy.*—Not so quickly. There is enough free acid in the peroxide of hydrogen to break up the old tract; what is not encysted is thrown out in two or three days.

*Dr. Brackett.*—Does Dr. Eddy insert the solution of gutta-percha immediately after cleansing the root?

*Dr. Eddy.*—I do where a fistula exists.

*Dr. Grant.*—I would like to ask Dr. Eddy if he can say whether there is an antiseptic property in chloroform?

*Dr. Eddy.*—I cannot say. I use chloroform to dissolve the gutta-percha, and the mass of chloro-percha is mixed with an equal proportion of an essential oil, such as eucalyptus. The substance then becomes of such a nature as to readily stick to the walls of the cavity.

*Dr. Grant.*—My idea is that chloroform is an antiseptic. I have sometimes thought that the chloroform had as much to do with the success of this treatment as the previous antiseptic applications.

*Dr. Eddy.*—There is hardly any chloroform in the solution that I use. It is evaporated. My method is to dissolve gutta-percha to the consistency of thick cream, dip an old Donaldson's broach into the solution, and fill not only the canal but the whole pulp-chamber. Then with a piece of unvulcanized rubber and a blunt instrument, used as a piston, force the solution of gutta-percha through the canal and fistulous opening. If there is no fistulous opening, then smear the walls of the canal with the solution, and at once carry a hard cone of gutta-percha to position.

*Dr. Clapp.*—Without causing considerable pain, I have had the greatest difficulty in getting creamy substances into such fine roots as have not a fistulous opening. Air prevents the solution from going in, and I feel that the chances of forcing gutta-percha to the end of the root are very slight. The only way I can satisfactorily accomplish this result is by first filling the root with some essential oil, either eucalyptus or some other, and following that with the solution of gutta-percha. Capillary attraction will assist in carrying it in. If I attempt to put chloro-percha into the root without first using an essential oil, I feel that I fail almost every time.

*Dr. Briggs.*—The gutta-percha which I use is pure, not that which we use for stopping, which may be gutta-percha and oxide of zinc and some other substance, but simply the pure gum dissolved in chloroform, with the essential oil added.

*Dr. Eddy.*—I use the same thing; it is about seal-brown in color.

*President Brackett.*—Where is this pure gutta-percha obtained?

*Dr. Eddy.*—In any rubber-store.

*Dr. Banfield.*—Will Dr. Eddy please state just the proportions in the mixture?

*Dr. Eddy.*—There are no special proportions.

*Dr. Eames.*—I am in accord with what has been said in regard to the efficiency of gutta-percha for filling canals, and would say that I am very much indebted to Dr. Clapp for showing me how to fill canals in a way similar to that described.

I have very frequently noticed the fact that pain has been occasioned by forcing the filling material into a canal, and I attributed it, as Dr. Clapp has, to the pressure of air within the canal. When such has been the case, I take a much finer instrument and fill less rapidly.

To give compactness to the filling, I have been in the habit for three or four years of forcing gold and platinum wire, and more recently copper wire, into the gutta-percha mass, and leaving it there.

*Dr. Eddy.*—I have a patient who has a tooth, the root of which was filled with cedar-wood by Dr. Seabury twenty years ago, and it is in just as good condition to-day as it was when filled.

*Dr. Seabury.*—I have used cedar-wood for more than thirty-five years, and I think my success is, perhaps, as uniform as anybody's. It is peculiarly fitted for filling root-canals, as it is very soft laterally and adapts itself readily to any irregularity in the canal, and is very firm when dried. I must say I am a little surprised at the success of driving gutta-percha up through a tooth. I should expect that it would make trouble. I never dared to fill a tooth with gold when I knew it was open at the apex.

